# Network Neuroanatomy of Self-Awareness: A Narrative Review of Self-Referential Processing, Metacognition, and Embodiment

**DOI:** 10.7759/cureus.116848

**Published:** 2026-09-24

**Authors:** Angelos Kandilas, Alexandros Samolis, Theodosis Kalamatianos, Ioannis Margaritis, George Tsakotos, George Triantafyllou, Maria Piagkou

**Affiliations:** 1 Department of Anatomy, School of Medicine, Faculty of Health Sciences, National and Kapodistrian University of Athens, Athens, GRC; 2 Department of Biomedical Engineering, University of West Attica, Athens, GRC

**Keywords:** anosognosia, bodily self-consciousness, default mode network, depersonalization, metacognition, self-awareness, self-referential processing

## Abstract

Self-awareness encompasses the capacity to recognize and evaluate information about oneself, including mental states, actions, autobiographical history, and bodily experience. Although closely related to consciousness, autobiographical memory, metacognition, clinical insight, anosognosia, and bodily self-consciousness, these constructs are not interchangeable. This focused narrative review synthesizes human neuroanatomical and neuroimaging evidence identified through a targeted PubMed/MEDLINE search covering January 1997 to August 15, 2026, supplemented by reference-chain searching. To improve methodological transparency, the revised review specifies eligibility principles, the narrative synthesis strategy, and reproducible domain-specific Boolean search strings; because the article is a narrative rather than a systematic review, no protocol registration, PRISMA screening flow, formal study-level risk-of-bias assessment, or quantitative pooling was undertaken. The evidence is organized into four interacting domains: self-referential representation, autobiographical continuity, metacognitive monitoring, and embodied self-processing. Cortical midline and default mode regions are prominent in self-referential evaluation; medial temporal systems support autobiographical information; anterior and lateral prefrontal and frontoparietal regions contribute to monitoring and updating; and temporoparietal, precuneal, insular, and sensorimotor regions contribute to bodily self-processing. Importantly, these macroscopic territories overlap and contain functionally heterogeneous subregions, so the proposed mappings represent network-weighted tendencies rather than one-to-one anatomical assignments. Clinical findings further show that anosognosia in Alzheimer's disease, impaired insight in schizophrenia, altered self-experience in depersonalization-derealization disorder, and awareness deficits after acquired brain injury (ABI) involve different combinations of these processes. We therefore propose a representation-memory-monitoring-embodiment framework as a heuristic neuroanatomical synthesis, not a diagnostic or treatment model. Its value lies in separating constructs often grouped under the term self-awareness and in generating testable hypotheses for longitudinal multimodal imaging, lesion mapping, causal studies, and construct-matched interventions.

## Introduction and background

Self-awareness concerns the ability to recognize and evaluate information about oneself: what one is thinking or doing, what has happened in one's personal past, how accurately one is performing, and how one's body is experienced in space. It is related to consciousness but is narrower than the broader state of being awake and having subjective experience [1]. Several neighboring terms describe different operations. Autobiographical memory concerns personally experienced events; metacognition refers to judging one's own cognitive performance; clinical insight concerns recognition and appraisal of illness; anosognosia denotes impaired awareness of a neurological or cognitive deficit; and bodily self-consciousness includes body ownership, agency, self-location, and first-person perspective [2]. These constructs can coexist and interact, but findings from one should not automatically be generalized to another.

Modern neuroanatomical accounts therefore emphasize distributed networks rather than a single “self” center. The default mode network (DMN) comprises medial frontal, posterior cingulate/precuneal, lateral parietal, and temporal regions that are strongly engaged during internally directed cognition, including self-referential thought and autobiographical remembering [3-5]. The salience network (SN), centered on the anterior insular and cingulate regions, helps prioritize behaviorally relevant internal and external information, whereas frontoparietal control systems support monitoring, goal-directed adjustment, and flexible updating. These labels are useful organizing terms, not sharply bound anatomical units; their components vary across parcellation schemes and participate in multiple cognitive functions [6-44].

The present review also differs from earlier syntheses in scope. Northoff et al. primarily meta-analyzed self-referential processing [3]; major DMN reviews have characterized internally directed cognition more broadly [4,5]; Mograbi et al. provided a multidimensional cognitive-neuroscience framework for self-awareness [2]; and a 2026 review emphasized self-referential processing, empathy, and psychiatric interventions [28]. Our specific contribution is to place four non-equivalent operations-self-referential representation, autobiographical continuity, metacognitive monitoring, and embodiment-within a single neuroanatomical comparison and then examine how different combinations of these operations are expressed across neurodegenerative, psychotic, dissociative, and acquired brain injury (ABI) phenotypes. The aim is not to claim a universal self-awareness network but to make explicit where constructs converge, where they dissociate, and where apparently shared regions may represent different subregional or network-level processes.

Accordingly, we organize the literature around four recurrent domains: representation, memory, monitoring, and embodiment. The framework is intended as a construct-matching heuristic. It asks, for example, whether a reported “awareness” deficit primarily concerns self-relevance, access to autobiographical information, confidence/error monitoring and belief updating, or bodily self-experience. This distinction matters clinically because similar surface descriptions can reflect different neurocognitive mechanisms.

We purposively selected four clinical phenotypes rather than conducting an exhaustive diagnostic survey. They were chosen because they illustrate distinct self-awareness constructs: anosognosia for cognitive decline in Alzheimer’s disease (AD)/mild cognitive impairment (MCI); clinical or cognitive insight and metacognitive evaluation in schizophrenia; altered embodiment and self-experience with preserved reality testing in depersonalization-derealization disorder (DPDR); and offline, online/emergent, or anticipatory awareness after ABI. Other neurological and psychiatric conditions may also involve disturbances of self-related processing but fall outside this focused comparative scope.

## Review

Review methods: search strategy, eligibility, and narrative synthesis

This article is a focused critical narrative review, not a systematic or scoping review. The revised manuscript was strengthened by referencing the methodological principles emphasized by the Scale for the Assessment of Narrative Review Articles (SANRA), particularly an explicit statement of the review aim, a description of the literature search, appropriate referencing, and separation of evidence from interpretation [37]. We searched PubMed/MEDLINE for human literature published from January 1, 1997, through August 15, 2026. We chose this lower date boundary to emphasize the modern structural and functional neuroimaging era while retaining influential work relevant to the conceptual framework.

We organized searches by construct rather than by the umbrella term “self-awareness.” The four principal domain searches addressed: (1) self-referential processing; (2) autobiographical memory and continuity; (3) metacognition, confidence, and error monitoring; and (4) bodily self-consciousness, ownership, agency, and self-location. Additional searches addressed the four clinical phenotypes (AD/MCI anosognosia, schizophrenia insight/metacognition, DPDR, and ABI awareness deficits) and, during revision, construct-targeted intervention concepts including neuromodulation, metacognitive rehabilitation/training, and interoceptive interventions. Neuroanatomical and methodological terms included MRI/fMRI, structural imaging, connectivity, diffusion imaging, lesion studies, stimulation, DMN, SN, frontoparietal network (FPN), mPFC, PCC/precuneus, temporoparietal junction (TPJ), insula, hippocampus, and related terms. Full reproducible PubMed Boolean strings corresponding to these search concepts are provided in the Appendices. We also screened reference lists of recent reviews, meta-analyses, and key primary studies to identify influential or directly relevant publications not captured by a single wording of the search terms.

We restricted eligible evidence to human studies that explicitly linked a defined self-related construct to anatomical, functional, connectivity, lesion, or stimulation findings. We considered structural MRI, task and resting-state fMRI, diffusion/connectivity studies, lesion studies, stimulation studies, and relevant systematic reviews or meta-analyses. We excluded purely philosophical or theoretical papers, animal studies, and studies of global consciousness or arousal without an explicit self-related measure. Because “self-awareness,” insight, anosognosia, metacognition, and bodily self-consciousness are not interchangeable, we synthesized studies by the construct actually measured rather than by the terminology used by the original authors. We prioritized meta-analyses/systematic reviews, causal lesion or stimulation evidence, well-characterized neuroimaging studies, and landmark studies that clarified construct boundaries.

The evidence base was too heterogeneous for meaningful quantitative pooling. Studies differed in construct definition, populations, behavioral tasks and clinical scales, disease stage, imaging modality, preprocessing, and network parcellation. We therefore used a structured narrative synthesis: we first grouped findings into four functional domains, then compared them across the four purposively selected clinical phenotypes, and finally interpreted them for convergence, dissociation, anatomical overlap, and translational relevance. We did not apply a meta-analysis or a formal study-level risk-of-bias tool.

Because this was not an exhaustive systematic review, we did not prospectively register a protocol or present a PRISMA screening flow diagram. Presenting iterative, construct-specific narrative searching as a PRISMA screening pipeline would imply a level of exhaustive identification and selection that was not part of the study design. The absence of registration, formal risk-of-bias grading, and multi-database systematic screening is therefore treated as a limitation rather than obscured. The added search strings improve reproducibility of the search logic but do not eliminate the possibility of citation-selection bias.

A multidimensional network architecture of self-awareness

Self-Referential Representation and the Default Mode Network

Self-referential tasks repeatedly engage the medial prefrontal cortex (mPFC), posterior cingulate cortex (PCC)/precuneus, and additional lateral parietal and temporal regions [3-5]. Much of this anatomy overlaps with the DMN. This overlap is relevant, but it does not mean that the DMN is specific to self-awareness. The network is also active during autobiographical remembering, social cognition, future simulation, and other forms of internally focused cognition [4,5]. In self-related tasks, it may provide a context in which personally meaningful information links to memory and current goals. That interpretation also explains why self-referential processing should not be used as a synonym for self-awareness. Recognizing that information concerns oneself is different from remembering one's personal history, judging whether one's performance is accurate, or experiencing the body as one's own. These functions overlap, but each draws on additional neural systems.

Autobiographical Continuity and Medial Temporal-Memory Systems

A sense of personal continuity depends heavily on autobiographical memory. Past experiences contribute to current self-knowledge and also provide material for imagining one's future. Lesion and functional imaging studies implicate the hippocampus and the broader medial temporal lobe (MTL), together with prefrontal and posterior DMN regions, in this temporal aspect of self-related cognition [6].

MTL is therefore better viewed as a source of episodic and contextual information than as a discrete center for self-awareness. This distinction matters in neurodegenerative disease. Memory impairment and reduced awareness of cognitive decline often occur together, but one does not fully explain the other. A patient's awareness may also depend on whether older self-knowledge can be compared with present performance.

Metacognitive Monitoring and Frontoparietal Control Systems

Metacognition concerns judgments about one's own cognitive performance, such as confidence in a decision or recognition of an error. Functional imaging and lesion studies point to an important role for anterior and lateral prefrontal regions in metacognitive accuracy [7,8]. A coordinate-based meta-analysis further identified a domain-general network involving medial and lateral prefrontal cortex, precuneus, and insula during metacognitive judgments [30]. These regions form part of broader frontoparietal control systems that evaluate performance and modify behavior when circumstances or evidence change. This distinction is clinically useful. A person may retain a coherent self-representation yet be poor at judging whether it remains accurate. Impaired insight can therefore arise from failures of monitoring or updating even when self-referential processing is partly preserved. This helps explain why clinically similar descriptions of "reduced awareness" do not necessarily imply the same neurocognitive deficit.

Embodied Self: Temporoparietal, Precuneal, Insular, and Sensorimotor Systems

The self is also experienced through the body. Body ownership, self-location, first-person perspective, agency, and sensitivity to internal bodily signals are related but separable aspects of bodily self-consciousness. Multisensory studies implicate the TPJ in combining visual, vestibular, and somatosensory information relevant to self-location and perspective [9]. Interoceptive processing also contributes; experimentally altering bodily self-identification changes cortical responses to cardiac signals [10]. Causal stimulation studies have added the anterior precuneus to this picture, particularly for spatial and physical aspects of bodily self-processing [11]. Reviews of the field support a distributed account rather than a single anatomical locus [9-12,31]. The TPJ, precuneus, insula, somatosensory cortex, and premotor regions appear to contribute differently to multisensory integration, interoception, agency, and spatial representation of the body. These functions interact with autobiographical and metacognitive processes but should not be collapsed into them.

Network Coordination and Emerging Self-Awareness Network Models

Coordination among these systems is likely to be as important as activity within any one of them. The SN, particularly the anterior insula and anterior cingulate cortex (ACC), is well-placed to register behaviorally relevant and interoceptive signals while interacting with DMN and frontoparietal control systems. The role of anterior insular/SN regions in switching between default-mode and executive-control systems provides a plausible mechanism for this coordination [4,32]. This arrangement offers one route by which internally generated information about the self could be weighed against changing internal and external demands.

A recent study illustrates this shift toward network-level descriptions. Xia et al. constructed a candidate "self-awareness brain network" and examined its organization in schizophrenia and major depressive disorder [13]. The study supports the view that self-related processing depends on distributed connectivity rather than a single locus. However, the proposed network remains an emerging construct, and its anatomical boundaries and clinical specificity require independent replication.

Anatomical Overlap, Subregional Specificity, and Limits of Network Labels

The four domains should not be interpreted as four non-overlapping anatomical maps. Several recurring structures, particularly the precuneus/PCC and insula, are macroscopically broad regions containing subfields with different cytoarchitecture, connectivity, and network affiliations. Modern work on the precuneus emphasizes substantial internal organization rather than a unitary posteromedial node [11,44]. For example, stimulating a restricted anterior precuneal region can perturb bodily self-processing, and responsive sites may lie outside the canonical DMN while remaining reciprocally connected with it [11]. Other precuneal and PCC territories participate more strongly in self-referential, autobiographical, visuospatial, or memory-related networks. Thus, the label “precuneus” can refer to different functional territories across studies.

The insula is similarly heterogeneous. Cytoarchitectonic and connectivity-based accounts distinguish posterior/mid-insular territories more closely related to somatosensory and interoceptive representation from anterior insular territories more strongly associated with salience detection, affective integration, and large-scale network coordination [38]. Consequently, an “insula” finding in a bodily self-task is not necessarily anatomically or computationally equivalent to an anterior-insula finding in metacognitive or salience-related paradigms.

These distinctions also limit claims about directional communication. Most evidence reviewed here comes from task activation, resting-state association, structural covariance, or undirected functional connectivity; these methods do not by themselves establish the direction of information flow. Effective-connectivity analyses, intracranial stimulation, lesion mapping, and perturbational designs remain comparatively sparse. The representation-memory-monitoring-embodiment framework therefore maps constructs to network-weighted tendencies, not to exclusive nodes or fixed directional pathways. This qualification is central to interpreting the spatial overlap shown in Table 1.

**Table 1:** Proposed functional domains of self-awareness and their principal neural substrates. ACC: anterior cingulate cortex, DMN: default mode network, FPN: frontoparietal network, mPFC: medial prefrontal cortex, MTL: medial temporal lobe, PCC: posterior cingulate cortex, PFC: prefrontal cortex, TPJ: temporoparietal junction.

Domain	Core operation	Principal substrates	Representative evidence
Self-referential representation	Evaluation of information in relation to self	mPFC, PCC/precuneus, lateral parietal/temporal DMN regions	Meta-analysis and DMN studies [3-5]
Autobiographical continuity	Linking present self to personal past and future	Hippocampus/MTL with prefrontal and posterior DMN interactions	Lesion and functional neuroanatomy [6]
Metacognitive monitoring	Confidence, error monitoring, updating self-beliefs	Anterior/lateral PFC, ACC, FPN	Functional and lesion studies [7,8]
Embodied self	Self-location, perspective, ownership, agency, interoception	TPJ, anterior precuneus, insula, sensorimotor/premotor cortex	Multisensory and causal studies [9-12]

Clinical disorders: convergence without equivalence

Alzheimer's Disease and Anosognosia

Anosognosia in AD and MCI is not simply a consequence of forgetting. Earlier structural and functional studies implicated medial temporal, frontal, and cingulate regions in poor awareness of cognitive deficits [14-16,33]. Later work showed that patients with impaired awareness can also have abnormalities within DMN subnetworks involved in autobiographical and self-referential processing [17].

Recent resting-state work has provided a more detailed network account. In a 2024 study across the MCI-AD continuum, anosognosia was associated with altered connectivity involving DMN, SN, and FPN [18]. Complementing this network evidence, a recent multimodal Alzheimer’s Disease Neuroimaging Initiative analysis found that reduced cognitive awareness was associated with widespread amyloid burden and regional atrophy in MCI, while lower left posterior cingulate cortex metabolism was related to poorer awareness in both MCI and Alzheimer's disease [29]. Together, these findings suggest that anosognosia reflects stage-dependent interactions among disease pathology, self-referential systems, and monitoring networks rather than disruption of a single node.

The mechanism remains uncertain because most available studies are cross-sectional and correlational. Connectivity differences cannot establish that a particular network abnormality causes anosognosia. Even so, the combined evidence makes a purely mnemonic explanation difficult to sustain and suggests that awareness of decline depends partly on comparing current performance with established self-knowledge.

Schizophrenia: Self-Reflection, Insight, and Metacognition

In schizophrenia, studies labeled as investigations of "self-awareness" often measure clinical or cognitive insight. Those constructs differ from autobiographical identity and bodily self-consciousness. Poorer cognitive insight has been associated with prefrontal abnormalities, while other studies implicate the ACC, anterior prefrontal and parietal cortices, and white-matter connectivity [19,20]. Functional imaging also shows altered activity during self-evaluation and awareness tasks [21].

A systematic review and meta-analysis found that clinical and cognitive insight in psychotic disorders have distributed, partly distinct neural correlates [22]. The 2025 network study by Xia et al. adds further support for abnormalities across self-related systems in schizophrenia [13].

It is therefore more accurate to describe poor insight as a disturbance of particular self-evaluative processes than as a global loss of self-awareness. Depending on the patient and the measure used, the difficulty may involve self-referential valuation, metacognitive monitoring, attribution, or revision of beliefs about illness and performance.

Depersonalization-Derealization Disorder: Altered Rather Than Absent Selfhood

DPDR provides a different clinical example. Patients generally know the detachment feels subjective and unusual, even as they report estrangement from the self, body, or surroundings. Earlier functional imaging implicated medial prefrontal, insular, somatosensory, and temporoparietal regions involved in affective and bodily self-processing [23]. This pattern differs from the reduced awareness of deficits seen in anosognosia.

More recent studies have broadened the network picture. Jia et al. reported altered topology among self-referentially relevant regions in DPDR [24], whereas Zheng et al. found abnormalities in resting-state connectivity within and between large-scale networks in drug-naive patients [25].

These findings are compatible with disturbed integration of self-referential, affective, interoceptive, and sensorimotor information. They do not support a simple loss of consciousness or awareness. A systematic review of functional neuroimaging across dissociative disorders likewise emphasized recurrent prefrontal, cingulate, parietal, temporal, and insular abnormalities while underscoring the small and heterogeneous evidence base [34]. At present, none of these network findings should be treated as a disorder-specific biomarker.

Acquired Brain Injury as Complementary Lesion Evidence

ABI offers a complementary line of evidence because focal or diffuse tissue damage can affect monitoring systems more directly than functional imaging alone can show. In traumatic brain injury, impaired self-awareness has been associated with abnormal activity and connectivity in regions involved in performance monitoring and self-evaluation [26].

A systematic review of ABI studies found that different forms of impaired awareness were linked to partly different anatomical and functional abnormalities [27]. Anticipatory awareness was most often associated with frontal and white-matter damage, whereas online error processing and error recognition involved frontoparietal, cingulo-insular, default mode, and salience-related systems. This multidimensional interpretation is consistent with later work on the Dynamic Comprehensive Model of self-awareness and with recent proposals to distinguish declarative, online, and anticipatory awareness in severe ABI [35,36].

This variation matters when interpreting literature. Knowing that a deficit exists, noticing an error as it happens, anticipating future difficulty, and changing behavior afterward are related functions, but not the same process. Studies that use different definitions of awareness should therefore not be expected to identify identical neural correlates.

A Proposed Representation-Memory-Monitoring-Embodiment Organizing Framework

The evidence reviewed above can be arranged into four recurring functional domains: representation, memory, monitoring, and embodiment. We propose these domains as a heuristic organizing synthesis derived from the reviewed evidence, not as a validated anatomical taxonomy or newly established neurobiological model.

The representation domain refers mainly to cortical midline and DMN processes that mark information as self-relevant. The memory domain reflects the contribution of medial temporal structures and their interactions with the DMN to autobiographical and contextual continuity. Monitoring depends more heavily on anterior and lateral prefrontal, cingulate, salience, and frontoparietal systems that evaluate performance and revise self-related judgments. Embodiment draws on the TPJ, precuneus, insula, premotor cortex, and sensorimotor regions to integrate multisensory and interoceptive information about body ownership, agency, self-location, and perspective.

These domains are not anatomically independent. The same region may contribute to several operations, and the balance among networks changes with the task and clinical state. The proposed framework is therefore descriptive and hypothesis-generating: it separates functions often grouped under the same label and makes it easier to compare findings across disorders without implying four discrete cerebral modules. Figure 1 summarizes this proposed organizing framework.

**Figure 1:**
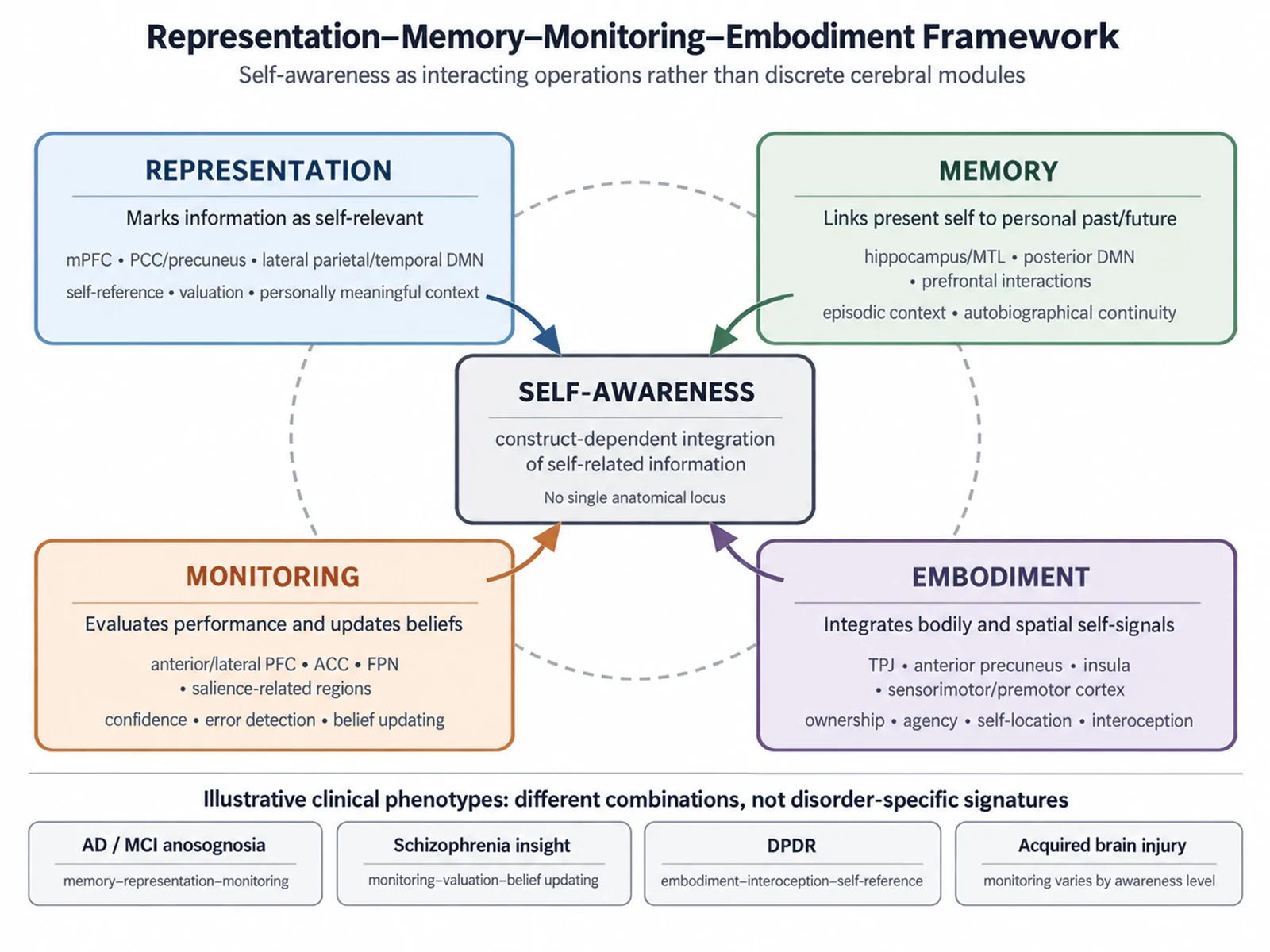
Proposed representation-memory-monitoring-embodiment framework for self-awareness. mPFC: medial prefrontal cortex, PCC: posterior cingulate cortex, MTL: medial temporal lobe, PFC: prefrontal cortex, ACC: anterior cingulate cortex, FPN: frontoparietal network, TPJ: temporoparietal junction, AD: Alzheimer's disease, MCI: mild cognitive impairment, DPDR: depersonalization-derealization disorder. Image Credits: This figure was created by the authors using DALL·E (OpenAI, San Francisco, CA, USA) via ChatGPT.

The four domains are presented as interacting operations rather than anatomically independent modules. Overlapping connectors indicate that the same macroscopic region or network may contribute to more than one operation, while the clinical phenotypes illustrate different disturbance patterns rather than disorder-specific imaging signatures.

Across diagnoses, the framework highlights distinct patterns rather than a single transdiagnostic lesion. In AD, anosognosia may involve weak integration between autobiographical/self-referential information and monitoring. In schizophrenia, difficulties may be more apparent in metacognitive evaluation and belief updating. In DPDR, embodied and affective aspects of the self-appear particularly relevant. After ABI, the phenotype varies depending on which monitoring process or network interaction has been disrupted.

These interpretations are hypotheses derived from heterogeneous literature. The disorders differ in pathology, symptoms, study design, and the measures used to define self-awareness. Direct testing with comparable tasks, multimodal imaging, lesion mapping, and longitudinal designs are needed before the proposed domains can support disorder-specific or causal inference.

Table 2 compares the major clinical phenotypes and their predominant network-level interpretations.

**Table 2:** Clinical phenotypes and dominant network-level interpretations. ACC: anterior cingulate cortex, DMN: default mode network, DPDR: depersonalization-derealization disorder, FPN: frontoparietal network, MCI: mild cognitive impairment, MTL: medial temporal lobe, SN: salience network, TPJ: temporoparietal junction.

Condition	Awareness phenotype	Dominant neural findings	Interpretation
Alzheimer's disease/MCI	Anosognosia for cognitive deficits	DMN, SN, and FPN dysconnectivity; cingulate/MTL contributions and multimodal biomarker associations [14-18,29]	Proposed impairment in integrating stored self-knowledge with monitoring of current performance
Schizophrenia	Poor clinical/cognitive insight; altered self-evaluation	Prefrontal, ACC, parietal, and distributed self-awareness network abnormalities [13,19-22]	Possible impairment of self-evaluation and belief updating; findings depend on the insight construct measured
DPDR	Detachment from self/body with retained reality testing	Self-referential, insular, sensorimotor, TPJ, and large-scale network alterations [23-25]	Disturbed embodiment and integration rather than global absence of self-awareness
Acquired brain injury	Offline, emergent, or anticipatory awareness deficits	Frontal/white-matter, ACC-insular-FPN, and DMN-SN abnormalities [26,27]	Different awareness levels may depend on partly distinct monitoring processes

Clinical implications, construct-matched interventions, and interpretive cautions

From a clinical perspective, the proposed organizing framework is most useful as a construct-matching tool. Anosognosia, poor clinical insight, depersonalization, and impaired online error awareness may all be described informally as disturbances of self-awareness, yet they should not be treated as equivalent symptoms or expected to share a single imaging signature. Clinical assessment should therefore specify whether the problem concerns knowledge of a deficit, confidence in performance, belief updating, autobiographical self-knowledge, agency, body ownership, interoception, or another component.

The same principle applies to intervention. A network association does not by itself establish a therapeutic target, and evidence for construct-specific neuromodulation remains preliminary. In DPDR, a small early clinical trial of right TPJ repetitive transcranial magnetic stimulation (rTMS) reported symptomatic improvement in a subset of 12 patients, supporting the feasibility of perturbing a region implicated in bodily self-processing but not establishing a validated network-targeted treatment [39]. Experimental transcranial direct current stimulation (tDCS) studies of metacognition likewise illustrate why anatomical targeting should not be equated with selective modulation of a self-awareness construct: stimulation of prefrontal, superior medial frontal, or posterior parietal sites altered decision-related processes in one recent study but produced little to no evidence of improved metacognitive performance [43]. Such findings argue for behavioral construct validation alongside any stimulation endpoint.

Psychological and rehabilitation approaches currently have a broader evidence base than node-specific stimulation for some phenotypes. Metacognitive training in psychosis targets cognitive biases and belief evaluation; a recent systematic meta-review found consistent evidence for improvement in several psychosis-related outcomes, although methodological quality and the specificity of mechanisms remain important limitations [40]. In ABI, a 2025 systematic review and meta-analysis found overall improvement from self-awareness interventions and suggested that interventions incorporating metacognitive techniques may be particularly useful, while also identifying substantial heterogeneity and possible publication bias [41]. For embodiment and interoception, psychotherapeutic approaches that explicitly work with interoceptive processes are developing, but a 2026 systematic review concluded that definitions, measurement, and treatment protocols remain fragmented [42].

These intervention literatures should therefore be viewed as translational tests of construct specificity rather than confirmation of a one-node/one-symptom model. The proposed framework may help future trials match the intervention, behavioral endpoint, and anatomical hypothesis, for example, distinguishing confidence calibration from clinical insight or body ownership from global distress, but it is not a treatment-selection algorithm. Within the evidence reviewed here, no imaging or stimulation signature has sufficient validation to guide individual diagnosis, prognosis, or personalized neuromodulation.

Neuroimaging findings should likewise be interpreted at the group and mechanism level rather than as individual diagnostic biomarkers. DMN, SN, FPN, medial temporal, temporoparietal, precuneal, and insular abnormalities are each implicated in multiple cognitive and clinical states. Their current value is primarily explanatory and hypothesis-generating; clinical translation requires prospective replication with standardized, construct-specific behavioral measures and explicit tests of whether changing the proposed mechanism changes the relevant awareness phenotype.

Limitations and future directions

Several limitations affect both the literature and this review. The first is conceptual. “Self-awareness” is used for tasks as different as trait judgments, confidence ratings, error detection, clinical insight, anosognosia, body ownership, agency, and depersonalization. Results from these paradigms may not reflect a single neurocognitive process, and differences in terminology likely contribute to apparent anatomical inconsistency across studies.

A second limitation concerns the evidence itself. Much of the clinical neuroimaging literature is cross-sectional, frequently involves modest samples, and varies in disease stage, medication exposure, behavioral assessment, image acquisition, preprocessing, and network parcellation. Such studies can identify associations but cannot determine whether a network difference precedes altered self-awareness, follows it, or reflects compensation. Network labels are also not anatomically invariant. Macroscopic terms such as “precuneus” and “insula” may collapse distinct cytoarchitectonic and connectivity-defined territories, while DMN, SN, and FPN boundaries differ across analytical schemes. Directional effective-connectivity and perturbational evidence remain much less abundant than correlational functional-connectivity evidence.

This review has methodological constraints inherent to its narrative design. The search relied mainly on PubMed/MEDLINE and reference-chain searching rather than systematic screening of multiple databases such as Embase, Scopus, Web of Science, and PsycINFO. Psychiatric, psychological, rehabilitation, and European literature that is incompletely indexed in PubMed may therefore be underrepresented. Study selection was relevance-driven rather than an exhaustive, independently duplicated screening process; no prospective protocol was registered; and no formal study-level risk-of-bias instrument or evidence-grading system was applied. These features increase vulnerability to citation-selection bias. Providing the domain-specific Boolean strings in the Appendices improves reproducibility of the search logic but does not convert the review into a systematic review or remove those limitations. The framework should therefore be read as an interpretation of convergent and dissociable findings, not as a complete catalog, quantitative evidence hierarchy, or prevalence estimate.

Future studies would benefit from defining the component of self-awareness before data collection and matching the behavioral measure to that construct. Multimodal designs that combine structural MRI or diffusion imaging with resting-state and task fMRI could help separate regional damage from altered communication between networks. Report high-resolution parcellation where possible so findings attributed to the precuneus, insula, medial prefrontal cortex, or TPJ can be localized more precisely. Effective-connectivity analyses, lesion-symptom mapping, and, where ethically and clinically appropriate, non-invasive or intracranial stimulation can provide stronger tests of causal and directional hypotheses.

Longitudinal and cross-disorder studies are particularly needed. Following patients over time may clarify whether changes in self-related networks precede, accompany, or follow changes in awareness. Using comparable tasks across AD, schizophrenia, DPDR, and ABI would help determine which mechanisms are shared and which are diagnosis-specific. Intervention studies should also prespecify whether they aim to change symptom severity, metacognitive sensitivity, confidence bias, insight, error awareness, body ownership, interoception, or another construct; mechanistic imaging endpoints are most informative when paired with a behavioral change in the same construct. Network measures may eventually aid phenotyping or rehabilitation planning, but this will require replication, standardized assessment, external validation, and demonstration of clinically meaningful prediction at the individual-patient level.

## Conclusions

No single cerebral region can explain self-awareness. The literature instead points to interacting systems that contribute differently to self-referential representation, autobiographical continuity, metacognitive monitoring, and bodily self-experience. Cortical midline and DMN regions are prominent during self-related evaluation; medial temporal structures supply autobiographical information; prefrontal and frontoparietal systems contribute to monitoring and updating; and temporoparietal, precuneal, insular, and sensorimotor regions support aspects of embodiment. However, these macroscopic territories contain functionally heterogeneous subregions and participate in multiple networks, so spatial overlap should not be mistaken for functional equivalence. Salience-related regions may help coordinate relevant signals across systems, but directional causal evidence remains limited. Clinical observations reinforce this multidimensional view. Anosognosia in AD, poor insight in schizophrenia, depersonalization-derealization, and awareness deficits after ABI are distinct phenomena involving different combinations of self-related processes. The proposed representation-memory-monitoring-embodiment framework is therefore intended as a construct-matching and hypothesis-generating heuristic, not as a new diagnostic taxonomy, imaging biomarker, or treatment algorithm. Its usefulness will depend on construct-specific behavioral measures, higher-resolution anatomical reporting, longitudinal multimodal imaging, lesion mapping, effective connectivity and perturbational methods, and intervention studies that demonstrate that altering a proposed mechanism changes the corresponding self-awareness phenotype.

## Appendices

Reproducible PubMed/MEDLINE search strategy

Database: PubMed/MEDLINE Coverage used for the review: January 1, 1997, to August 15, 2026 Population: Humans Purpose: These strings operationalize the construct-specific search logic used for the narrative synthesis. They are provided to improve reproducibility of the search logic; they are not presented as an exhaustive systematic-review protocol, and no PRISMA screening counts are inferred from them.

For each search below, the following date/human filter may be appended:

AND ("1997/01/01"[Date - Publication] : "2026/08/15"[Date - Publication]) AND Humans[MeSH Terms]

A. Functional-domain searches

1. Self-referential representation

("self-awareness"[Title/Abstract] OR "self-referential processing"[Title/Abstract] OR "self-related processing"[Title/Abstract] OR "self-reference"[Title/Abstract]) AND ("neuroanatomy"[Title/Abstract] OR neuroimaging[Title/Abstract] OR MRI[Title/Abstract] OR fMRI[Title/Abstract] OR connectivity[Title/Abstract] OR lesion*[Title/Abstract] OR stimulation[Title/Abstract] OR "default mode network"[Title/Abstract] OR "medial prefrontal cortex"[Title/Abstract] OR "posterior cingulate cortex"[Title/Abstract] OR precuneus[Title/Abstract])

2. Autobiographical continuity

("autobiographical memory"[Title/Abstract] OR "autobiographical self"[Title/Abstract] OR "personal memory"[Title/Abstract] OR "self-knowledge"[Title/Abstract]) AND (hippocamp*[Title/Abstract] OR "medial temporal"[Title/Abstract] OR "default mode network"[Title/Abstract] OR "posterior cingulate"[Title/Abstract] OR precuneus[Title/Abstract] OR "medial prefrontal"[Title/Abstract] OR neuroimaging[Title/Abstract] OR lesion*[Title/Abstract] OR connectivity[Title/Abstract])

3. Metacognitive monitoring

(metacognition[Title/Abstract] OR metacognitive[Title/Abstract] OR confidence[Title/Abstract] OR "error monitoring"[Title/Abstract] OR "performance monitoring"[Title/Abstract]) AND ("prefrontal cortex"[Title/Abstract] OR "anterior prefrontal"[Title/Abstract] OR "frontoparietal network"[Title/Abstract] OR "anterior cingulate"[Title/Abstract] OR insula[Title/Abstract] OR precuneus[Title/Abstract] OR neuroimaging[Title/Abstract] OR lesion*[Title/Abstract] OR stimulation[Title/Abstract])

4. Embodied self-processing

("bodily self-consciousness"[Title/Abstract] OR "body ownership"[Title/Abstract] OR "self-location"[Title/Abstract] OR "first-person perspective"[Title/Abstract] OR agency[Title/Abstract] OR interoception[Title/Abstract] OR "bodily self"[Title/Abstract]) AND ("temporoparietal junction"[Title/Abstract] OR TPJ[Title/Abstract] OR precuneus[Title/Abstract] OR insula[Title/Abstract] OR sensorimotor[Title/Abstract] OR premotor[Title/Abstract] OR neuroimaging[Title/Abstract] OR stimulation[Title/Abstract] OR lesion*[Title/Abstract])

B. Clinical-phenotype searches

5. Alzheimer's disease/MCI and anosognosia

("Alzheimer disease"[Title/Abstract] OR "Alzheimer's disease"[Title/Abstract] OR "mild cognitive impairment"[Title/Abstract] OR MCI[Title/Abstract]) AND (anosognosia[Title/Abstract] OR "impaired awareness"[Title/Abstract] OR "cognitive awareness"[Title/Abstract] OR "self-awareness"[Title/Abstract]) AND (MRI[Title/Abstract] OR fMRI[Title/Abstract] OR PET[Title/Abstract] OR connectivity[Title/Abstract] OR "default mode network"[Title/Abstract] OR "salience network"[Title/Abstract] OR "frontoparietal network"[Title/Abstract] OR cingulate[Title/Abstract] OR hippocamp*[Title/Abstract])

6. Schizophrenia, insight, and metacognition

(schizophrenia[Title/Abstract] OR psychosis[Title/Abstract] OR psychotic[Title/Abstract]) AND ("clinical insight"[Title/Abstract] OR "cognitive insight"[Title/Abstract] OR insight[Title/Abstract] OR metacognition[Title/Abstract] OR "self-evaluation"[Title/Abstract] OR "self-awareness"[Title/Abstract]) AND (neuroimaging[Title/Abstract] OR MRI[Title/Abstract] OR fMRI[Title/Abstract] OR connectivity[Title/Abstract] OR "prefrontal cortex"[Title/Abstract] OR cingulate[Title/Abstract] OR insula[Title/Abstract] OR parietal[Title/Abstract])

7. Depersonalization-derealization disorder

(depersonalization[Title/Abstract] OR derealization[Title/Abstract] OR "depersonalization-derealization disorder"[Title/Abstract] OR DPDR[Title/Abstract]) AND ("self-referential"[Title/Abstract] OR embodiment[Title/Abstract] OR interoception[Title/Abstract] OR "body ownership"[Title/Abstract] OR "self-awareness"[Title/Abstract]) AND (neuroimaging[Title/Abstract] OR MRI[Title/Abstract] OR fMRI[Title/Abstract] OR connectivity[Title/Abstract] OR insula[Title/Abstract] OR "temporoparietal junction"[Title/Abstract] OR precuneus[Title/Abstract] OR sensorimotor[Title/Abstract])

8. Acquired brain injury and awareness

("acquired brain injury"[Title/Abstract] OR "traumatic brain injury"[Title/Abstract] OR TBI[Title/Abstract] OR stroke[Title/Abstract]) AND ("impaired self-awareness"[Title/Abstract] OR "self-awareness"[Title/Abstract] OR anosognosia[Title/Abstract] OR "error awareness"[Title/Abstract] OR "online awareness"[Title/Abstract] OR "anticipatory awareness"[Title/Abstract] OR "emergent awareness"[Title/Abstract]) AND (lesion*[Title/Abstract] OR neuroimaging[Title/Abstract] OR fMRI[Title/Abstract] OR connectivity[Title/Abstract] OR frontal[Title/Abstract] OR cingulate[Title/Abstract] OR insula[Title/Abstract] OR frontoparietal[Title/Abstract] OR "white matter"[Title/Abstract])

C. Targeted intervention searches added for the revision

9. Neuromodulation

("self-awareness"[Title/Abstract] OR metacognition[Title/Abstract] OR insight[Title/Abstract] OR anosognosia[Title/Abstract] OR depersonalization[Title/Abstract] OR "bodily self"[Title/Abstract]) AND (rTMS[Title/Abstract] OR "repetitive transcranial magnetic stimulation"[Title/Abstract] OR tDCS[Title/Abstract] OR "transcranial direct current stimulation"[Title/Abstract] OR neuromodulation[Title/Abstract] OR stimulation[Title/Abstract])

10. Metacognitive/rehabilitation interventions

("self-awareness"[Title/Abstract] OR metacognition[Title/Abstract] OR insight[Title/Abstract]) AND ("metacognitive training"[Title/Abstract] OR "metacognitive strategy"[Title/Abstract] OR rehabilitation[Title/Abstract] OR feedback[Title/Abstract] OR psychoeducation[Title/Abstract] OR intervention[Title/Abstract]) AND (schizophrenia[Title/Abstract] OR psychosis[Title/Abstract] OR "acquired brain injury"[Title/Abstract] OR "traumatic brain injury"[Title/Abstract])

11. Interoceptive interventions

(interoception[Title/Abstract] OR interoceptive[Title/Abstract]) AND (therapy[Title/Abstract] OR psychotherapy[Title/Abstract] OR intervention[Title/Abstract] OR training[Title/Abstract] OR feedback[Title/Abstract]) AND ("self"[Title/Abstract] OR embodiment[Title/Abstract] OR depersonalization[Title/Abstract] OR dissociation[Title/Abstract])

Search Interpretation

The review used these searches as construct-specific discovery tools rather than as one universal query. We used reference-chain searching after identifying key systematic reviews, meta-analyses, causal lesion/stimulation studies, and landmark neuroimaging papers. Because the review was not designed as a systematic review, we did not use a prospective deduplication/screening pipeline, register a protocol, include a PRISMA flow diagram, or conduct a formal study-level risk-of-bias assessment. The revised manuscript explicitly acknowledges these methodological constraints.

## References

[1] Zeman A (2001). Consciousness. Brain.

[2] Mograbi DC, Hall S, Arantes B, Huntley J (2024). The cognitive neuroscience of self-awareness: Current framework, clinical implications, and future research directions. Wiley Interdiscip Rev Cogn Sci.

[3] Northoff G, Heinzel A, de Greck M, Bermpohl F, Dobrowolny H, Panksepp J (2006). Self-referential processing in our brain--a meta-analysis of imaging studies on the self. Neuroimage.

[4] Menon V (2023). 20 years of the default mode network: a review and synthesis. Neuron.

[5] Davey CG, Pujol J, Harrison BJ (2016). Mapping the self in the brain's default mode network. Neuroimage.

[6] Levine B (2004). Autobiographical memory and the self in time: brain lesion effects, functional neuroanatomy, and lifespan development. Brain Cogn.

[7] Fleming SM, Huijgen J, Dolan RJ (2012). Prefrontal contributions to metacognition in perceptual decision making. J Neurosci.

[8] Fleming SM, Ryu J, Golfinos JG, Blackmon KE (2014). Domain-specific impairment in metacognitive accuracy following anterior prefrontal lesions. Brain.

[9] Ionta S, Heydrich L, Lenggenhager B (2011). Multisensory mechanisms in temporo-parietal cortex support self-location and first-person perspective. Neuron.

[10] Park HD, Bernasconi F, Bello-Ruiz J, Pfeiffer C, Salomon R, Blanke O (2016). Transient modulations of neural responses to heartbeats covary with bodily self-consciousness. J Neurosci.

[11] Lyu D, Stieger JR, Davey WP (2023). Causal evidence for the processing of bodily self in the anterior precuneus. Neuron.

[12] Dary Z, Lopez C (2023). Understanding the neural bases of bodily self-consciousness: recent achievements and main challenges. Front Integr Neurosci.

[13] Xia X, Gao F, Xu S (2025). The self-awareness brain network: construction, characterization, and alterations in schizophrenia and major depressive disorder. Neuroimage.

[14] Chavoix C, Insausti R (2017). Self-awareness and the medial temporal lobe in neurodegenerative diseases. Neurosci Biobehav Rev.

[15] Zamboni G, Drazich E, McCulloch E (2013). Neuroanatomy of impaired self-awareness in Alzheimer's disease and mild cognitive impairment. Cortex.

[16] Amanzio M, Torta DM, Sacco K (2011). Unawareness of deficits in Alzheimer's disease: role of the cingulate cortex. Brain.

[17] Antoine N, Bahri MA, Bastin C (2019). Anosognosia and default mode subnetwork dysfunction in Alzheimer's disease. Hum Brain Mapp.

[18] Tondelli M, Ballotta D, Maramotti R (2024). Resting-state networks and anosognosia in Alzheimer's disease. Front Aging Neurosci.

[19] Orfei MD, Piras F, Macci E, Caltagirone C, Spalletta G (2013). The neuroanatomical correlates of cognitive insight in schizophrenia. Soc Cogn Affect Neurosci.

[20] Spalletta G, Piras F, Piras F, Caltagirone C, Orfei MD (2014). The structural neuroanatomy of metacognitive insight in schizophrenia and its psychopathological and neuropsychological correlates. Hum Brain Mapp.

[21] Shad MU, Keshavan MS, Steinberg JL (2012). Neurobiology of self-awareness in schizophrenia: an fMRI study. Schizophr Res.

[22] Pijnenborg GH, Larabi DI, Xu P (2020). Brain areas associated with clinical and cognitive insight in psychotic disorders: a systematic review and meta-analysis. Neurosci Biobehav Rev.

[23] Medford N, Sierra M, Stringaris A, Giampietro V, Brammer MJ, David AS (2016). Emotional experience and awareness of self: functional MRI studies of depersonalization disorder. Front Psychol.

[24] Jia Y, Song N, Ning Y (2025). Altered self-referential-related brain regions in depersonalization-derealization disorder. Brain Behav.

[25] Zheng S, Song M, Song N (2025). Dysfunctional large-scale brain networks in drug-naïve depersonalization-derealization disorder patients. BMC Psychiatry.

[26] Ham TE, Bonnelle V, Hellyer P, Jilka S, Robertson IH, Leech R, Sharp DJ (2014). The neural basis of impaired self-awareness after traumatic brain injury. Brain.

[27] Terneusen A, Winkens I, van Heugten C, Stapert S, Jacobs HI, Ponds R, Quaedflieg C (2023). Neural correlates of impaired self-awareness of deficits after acquired brain injury: a systematic review. Neuropsychol Rev.

[28] Siqintu, Bao Q, Yang D (2026). Disrupted self-referential processing and empathy-based interventions in mental disorders: neural mechanisms, cognitive biases, and therapeutic integration. Front Psychiatry.

[29] Cacciamani F, Houot M, Tezenas du Montcel S, Thibeau-Sutre E, Vannini P, Migliaccio RL (2026). Multimodal neural correlates of cognitive awareness in aging and Alzheimer's disease. NeuroImage Clin.

[30] Vaccaro AG, Fleming SM (2018). Thinking about thinking: a coordinate-based meta-analysis of neuroimaging studies of metacognitive judgements. Brain Neurosci Adv.

[31] Ronchi R, Park HD, Blanke O (2018). Bodily self-consciousness and its disorders. Handb Clin Neurol.

[32] Sridharan D, Levitin DJ, Menon V (2008). A critical role for the right fronto-insular cortex in switching between central-executive and default-mode networks. Proc Natl Acad Sci U S A.

[33] Hallam B, Chan J, Gonzalez Costafreda S, Bhome R, Huntley J (2020). What are the neural correlates of meta-cognition and anosognosia in Alzheimer's disease? A systematic review. Neurobiol Aging.

[34] Modesti MN, Rapisarda L, Capriotti G, Del Casale A (2022). Functional neuroimaging in dissociative disorders: a systematic review. J Pers Med.

[35] Toglia J, Goverover Y (2022). Revisiting the dynamic comprehensive model of self-awareness: a scoping review and thematic analysis of its impact 20 years later. Neuropsychol Rehabil.

[36] Bivona U, Villalobos D (2025). Assessment of self-awareness after severe acquired brain injury: a systematic review and recommendations for a new classification of offline self-awareness. Neuropsychologia.

[37] Baethge C, Goldbeck-Wood S, Mertens S (2019). SANRA-a scale for the quality assessment of narrative review articles. Res Integr Peer Rev.

[38] Kittleson AR, Woodward ND, Heckers S, Sheffield JM (2024). The insula: leveraging cellular and systems-level research to better understand its roles in health and schizophrenia. Neurosci Biobehav Rev.

[39] Mantovani A, Simeon D, Urban N, Bulow P, Allart A, Lisanby S (2011). Temporo-parietal junction stimulation in the treatment of depersonalization disorder. Psychiatry Res.

[40] Meinhart A, Sauvé G, Schmueser A (2025). Metacognitive training for psychosis (MCT): a systematic meta-review of its effectiveness. Transl Psychiatry.

[41] Villalobos D, Bivona U, Botella J (2025). Self-awareness interventions after acquired brain injury: a systematic review and meta-analysis. Rehabil Psychol.

[42] Aquino I, Dufey M, Manríquez-Reyes K (2026). A systematic review of interoception and therapeutic outcome research: Implications for psychotherapeutic practice. Psychol Psychother.

[43] Sabio J, Ballard T, Filmer HL, Dux PE (2025). The influence of tDCS on the speed-accuracy tradeoff and metacognitive decision making. Neuropsychologia.

[44] Dadario NB, Sughrue ME (2023). The functional role of the precuneus. Brain.

